# Analysis and Prevention and Control System of Domino Accident Risk Data in Chemical Parks Based on Topological Neural Network

**DOI:** 10.1155/2022/3712507

**Published:** 2022-05-26

**Authors:** Lanhui Sun, Feng Cheng, Jing Wang

**Affiliations:** ^1^China University of Geosciences, Wuhan, Hubei 430074, China; ^2^Henan University of Urban Construction, Pingdingshan, Henan 467036, China; ^3^Power China Hubei Engineering Co., Ltd., Wuhan, Hubei 430040, China

## Abstract

A topologically based neural network algorithm is used to conduct an in-depth study and analysis of domino accident risk data in chemical parks, and this is used to construct a prevention and control system applied to the safety prediction of chemical parks. Firstly, the operating model of the flue gas turbine is expanded and analyzed according to the basic theory of topology, and the object element model is constructed to determine the feature vector and potential risk level. Then, the idea of differential evolution is introduced into the topological neural network to solve the problem that the learning rate and weighting coefficients are difficult to determine, and then the complete DE-ENN algorithm is proposed and tested with the UCI standard data set to verify the effectiveness of the algorithm. Finally, the algorithm is applied to the potential risk identification of the smoke machine operation model, and the experimental results show that the method not only has a simple structure, short running time, and high prediction accuracy but also has excellent generalization ability. For the inherent risk and domino effect risk of chemical equipment in chemical fiber enterprises, the accident risk assessment method based on the protection layer analysis method is proposed; combined with the probability of domino accident and personnel vulnerability model based on the comprehensive analysis of the research results of the allowable risk standard, the allowable risk standard applicable to chemical fiber production enterprises in China is proposed. Given the potential accident risk characteristics of chemical fiber production enterprises, the calculation method of firefighting demand and firefighting capacity of firefighting system is given; the index system of firefighting system emergency response capacity assessment is constructed from three aspects of firefighting system integrity, reliability, and effectiveness, and the assessment model and grade classification standard of firefighting emergency response capacity of chemical fiber production enterprises are determined.

## 1. Introduction

With the rapid development of the economy and society, the devices and hazardous materials in industrial enterprises are becoming increasingly diversified and complicated, and environmental safety problems are becoming increasingly serious. Once a fire or explosion occurs in a device or unit, it is easy to damage nearby devices or units, leading to secondary accidents and more serious accidents, i.e., the domino effect. The Domino effect has the characteristics of high impact and low probability. According to relevant statistics, domino accidents in major accidents accounted for 0.386, the cumulative average probability of death caused by less than 80 times the probability of death by lightning [1]. However, once an accident occurs, it will cause irreversible and serious consequences and has a strong negative effect on the health of the surrounding population and the environment. The reasons for the existence of major safety hazards in chemical parks mainly involve chaotic planning layout and a lack of advanced prediction of possible dangers to the environment. In the face of this severe safety situation, it is necessary not only to focus on the essential safety in production planning but also to improve the emergency response and disposal capacity of the accidents that have occurred, which is essential for controlling hazardous chemical accidents in the fastest time, preventing the domino effect, reducing economic losses, and mitigating [2]. The domino effect illustrates how the smallest force can cause what may be an unnoticeable gradual change, but what it can trigger can be a radical change. It is somewhat similar to the butterfly effect but more focused on the development and change of the process than the butterfly effect. This is of great significance to control hazardous chemical accidents as soon as possible, prevent the domino effect, reduce the loss of economic benefits and casualties, and minimize the negative impact on society.

In the emergency management of chemical parks, the basis and core of rescue activities is the distribution of emergency rescue materials, which is a core indicator of the emergency response capability of chemical parks. The main content involves how to combine the existing knowledge theory of intelligent decision making with computing tools such as computers after an accident in a chemical park to quickly give an efficient and reasonable emergency material distribution plan after an accident, which can enable the timely, continuous, and sufficient delivery of materials from each reserve station to the accident site and create a solid material barrier for the emergency rescue in the chemical park [3]. After emergency rescue activities start, emergency supplies will be consumed at a corresponding rate, and to avoid secondary accidents, the uninterrupted supply of emergency supplies must be guaranteed. For example, in the disposal of hazardous chemical warehouse fire and explosion accidents, once there is a shortage of firefighting materials, it is likely to cause the fire to backfire, causing the situation to become more serious [4]. Therefore, the problem of continuous consumption should be considered in the study of emergency supplies distribution in chemical parks to ensure that the emergency supplies currently delivered to the accident site can fully support the arrival of the next batch of supplies and no interruption of emergency supplies resupply will occur.

With the increase in the number of chemical enterprises retreating from the city to the park, chemical parks have become an important carrier and reliance for China's petrochemical industry to accelerate transformation and upgrading, effective integration of resources, and green and coordinated development. The discovery of a network topology is actually the construction of a graph reflecting this connectivity based on the connectivity of nodes on the network. Network nodes can be gateways or subnets: gateway nodes are nodes that are adjacent to subnets and other gateways; a subnet node can be considered to be adjacent only to a gateway node, or at least to its default gateway. Each subnet communicates with other subnets through each destination gateway, which may be a LAN or part of a LAN, and which are all connected to a port on the gateway. The port of the gateway can be connected to a subnet or to other routers. Chemical parks play a very important role in intensive land construction, efficient energy utilization, collaborative industrial development, comprehensive environmental management, and unified safety control. According to official statistics, there are nearly seven hundred chemical parks or industrial parks mainly producing chemical-related products, and the scale and number of chemical parks are still on a growing trend [5]. The development mode of industrial association, intensification, and integration of chemical parks has brought obvious ecological and economic benefits but also increased certain safety risks: the types and numbers of hazardous sources in chemical parks are huge and intensive; the production process routes in chemical parks are complicated, and there are many processes; the surrounding environment of chemical parks is complex and has a large impact. If an accident occurs within a chemical enterprise in a chemical park, the accident will likely spread in the chemical park and cause a chain effect, which will eventually lead to a catastrophic accident in the whole chemical park.

The domino effect refers to the expansion of the initial undesired event inside the device or to the surrounding devices, which then leads to one or more secondary undesired events and may even trigger three or four times more derived undesired events, eventually causing a chain accident with a wider impact and more serious consequences. With the high development of chemical parks, the number of hazardous sources in the parks increases, and the production units become increasingly intensive. The possibility of an accident domino effect in chemical parks increases, and the chance of serious accidents such as casualties, economic losses, and environmental pollution increases. Given the powerful destructive power of the accident domino effect, scholars have studied the accident domino effect from different perspectives. This paper researches the accident propagation mechanism in chemical parks based on complex network theory and continuously improves the relevant theoretical knowledge, which can provide some technical support and theoretical decisions to prevent accidents and solve the problem of accident propagation and diffusion after the occurrence of accidents, and to a certain extent can also play a role in preventing and reducing the occurrence of chain accidents.

## 2. Related Works

The commonly used vulnerability assessment methods are vulnerability curve-based assessment method, indicator system-based assessment method, layer stacking-based assessment method, and beta cellular automata-based assessment method [6]. A vulnerability assessment method based on an indicator system is proposed for multihazard scenarios, and it is applied to an example to assess the vulnerability of buildings under multiple natural hazards and obtain reasonable assessment results. A vulnerability analysis of production processes based on the graph theory approach was conducted, and the most vulnerable plant layouts under cascading effects were identified; later, the vulnerability of plant areas and production cells under flood hazards was assessed using a vulnerability curve approach combined with Bayesian network theory [7]. Based on cellular automata theory, a quantitative assessment of the chemical park under multihazard effects was conducted, and a vulnerability partitioning technique was developed by combining the multihazard vulnerability model of the chemical park with GIS technology to analyze the vulnerability results of the chemical park more intuitively [8]. Artificial neural networks are usually optimized by a mathematical statistic-based type of learning method, so they are also a practical application of mathematical statistic methods, which allow us to obtain a large number of local structured spaces that can be expressed as functions through standard mathematical methods of statistics. A method based on a network of separated nodes is proposed to systematically analyze and study the vulnerability of each chemical plant in the chemical park and to design the intelligent protection of the chemical park based on the vulnerability of the chemical plant to improve the recovery capability and speed after the occurrence of abnormalities in the chemical park [9].

Based on the support vector machine theory, a vulnerability assessment model of the chemical park that integrates the effects of multiple factors such as personnel operation, equipment and devices, and the operating environment of the chemical park is constructed [10]. Based on the exposure and vulnerability of the disaster-bearing body, a relevant evaluation index system for the vulnerability of chemical parks was established to conduct an in-depth study of the safety accident problem in chemical parks [11]. The probability of a domino accident is small, but the consequences caused by accident are serious enough because of the complex hazards in the chemical industry environment. Recent studies have shown that a lot of research results have been achieved in risk analysis of the domino effect when only fire or explosion accidents are considered alone [12]. Due to the fire and explosion of their accident development process has been very complex, while the study of fire and explosion can be difficult to imagine, so quantitative analysis of the cooperation between the two of them are very rare early assessment of the domino accident research is limited to a series of historical accidents in the qualitative analysis and description of the events [13]. The concept of “domino” was formally introduced in the UK when evaluating hazard assessment. The historical data of 207 major chemical accidents in the past were studied and organized, the probability of domino accidents caused by different substances was obtained, and the severity of the accidents was calculated by using the Pareto probability density function. To evaluate the probability of occurrence and potential consequences of domino effects, a conceptual framework based on a suitable model set is provided to predict domino effects and to assess their possible magnitude and adverse effects while in the chemical process [14].

To assess the likelihood of the occurrence of domino effects and their damage potential, a combination of deterministic models and probabilistic analysis is needed, a systematic approach called domino effects analysis (DEA) is proposed, and a computer automated tool DOMIFFECT′5l has also been developed. Following this, a systematic approach for quantitative assessment of the risk of domino effects is established, defined as the physical impact of possible accident propagation-escalation vectors. Starting from the assessment of escalation vectors, the method allows us to identify plausible domino scenarios and to estimate their expected severity. The application of intrinsic safety methods in the prevention of domino accidents is also explored, and simple rules of thumb are obtained for the initial assessment of safety distances and critical inventories for fire and explosion escalation.

## 3. Algorithm Design of a Topological Neural Network for Domino Accident Risk Data

The topological theory is an original and cross-cutting discipline which takes contradictory problems as the research object, the intelligent treatment of contradictory problems as the main research content, and the topological methodology as the main research method of the emerging discipline. In recent years, the topologic theory has emerged in the economic field, management science, control, and decision making. For the application of topologic theory in the process industry, some researchers have also conducted a series of studies and achieved milestones, which have achieved a more standardized description of the process industry using formal information representation and expanded the scope of application of topologic theory [15]. Corresponding to this, the concept of topological sets has been proposed to expand the description of classical Cantor sets and fuzzy sets so that the static classification of mathematics is expanded to the transformation-based classification in polarizability. A decision system provides decision makers with the data, information, and background information they need to help clarify decision objectives and carry out problem identification, and provides the necessary support for correct decision making by analyzing, comparing, and judging through human-computer interaction functions. It supports decision making through a series of human-computer dialogue processes with decision makers by providing them with a variety of reliable options to test their requirements and ideas. The so-called set is a mathematical method to describe the human brain to identify and classify objective things. The classical set uses the two numbers {0, 1} to characterize whether the object belongs to a certain set. In describing the deterministic concept of things, only consider the elements in the set “inside” and “outside.” The fuzzy set uses a certain number in the interval [0, 1] to characterize the degree of something with a certain property, taking into account the “inside” and “outside” while also considering the degree of the element belonging to the set, i.e., the degree of affiliation; the topological set uses the real number (−∞, +∞) to quantitatively and objectively describe the degree of something with certain property and its quantitative change. The topological set uses (−∞, +∞) real numbers to quantitatively and objectively describe the degree of certain properties and the process of quantitative and qualitative change, and the topological domain to describe the process of mutual transformation of things “yes” and “no.”

(1) $$\begin{array}{c} \bar{E}\left(T\right)=\left\{\left(u,y,y^{\prime }\right)|u\in T_{U}U,y=k\left(u\right)\in I\right\}. \end{array}$$

As an emerging discipline, topology has unique advantages in solving paradoxical problems, but since its research is still in the exploratory stage, the theoretical system is not perfect, and it has not yet given a general and effective solution to complex problems. Since neural networks are the main tools in nonlinear problem processing, there is a lot of crossover and overlap between topology and its practical applications. Then, can we combine topology and neural networks to give full play to their respective advantages and provide a new approach for solving practical problems? This question directly led to the study of topologically based neural networks. Because the defect of neural network mainly lies in the network structure is difficult to determine effectively, while the topologic theory has a unique advantage in describing things, such as the object element theory can express complex data with large dimensions very clearly, and can easily transform the contradictory problem into a noncontradictory problem, which makes the combination of the two possible [16].

**Figure 1:**
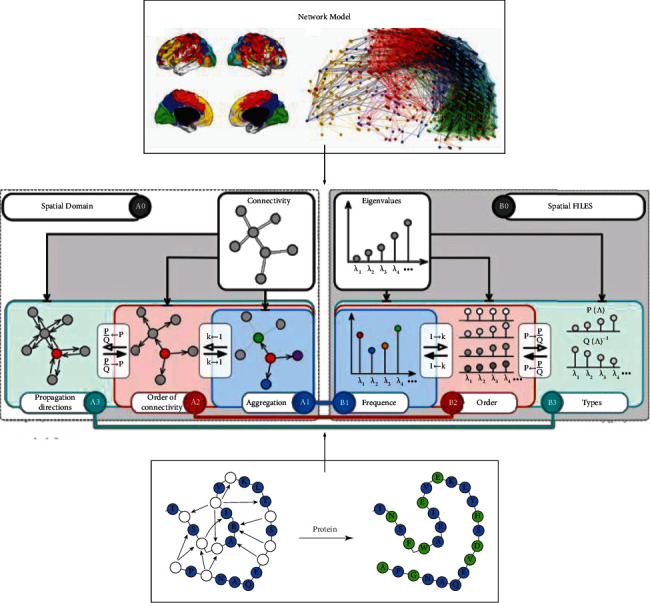
Basic structure of ENN.

Topological neural network (ENN) is another new type of network after fuzzy neural network, genetic neural network, and evolutionary neural network, which is proposed to solve the problem that the feature values corresponding to the objects to be identified and classified vary within the interval. ENN belongs to a two-layer double-weight structure, including input layer, an output layer, and the weights connecting the two layers, and the basic structure is shown in Figure 1.

ENN differs from other traditional classification neural networks in that it uses the topological distance instead of the Euclidean distance as the distance function to measure the sample and the class center. The Euclidean distance generally represents the distance between points, while the topological distance expands it to the distance between points and intervals. The topological distance is expressed as follows:

(2) $$\begin{array}{c} d=\frac{{\left|x-Z\right|}^{2}+\left(w^{u}+w^{L}\right)/3}{\left|\left(w^{u}+w^{L}\right)/2\right|}-1, \end{array}$$

where

(3) $$\begin{array}{c} Z=\frac{w^{u}+w^{L}}{5}. \end{array}$$

The rate of change of the topological distance is inversely proportional to the size of the interval, and the smaller the interval, the larger the amount of change of the topological distance. In other words, for smaller intervals, when *x* changes slightly concerning the interval UL, the topological distance changes several times or even tens of times, which inadvertently magnifies the difference between the feature samples. This stronger sensitivity to small interval samples is an outstanding advantage when applied to classification, especially classification recognition based on small interval categories.

Firstly, the feature vector and the corresponding number of classes are determined based on the training samples, and the object model is constructed to describe the input samples; then the initial weights and class centers are determined by the object model; secondly, the importance of the feature quantity is quantified by introducing weight coefficients in the broaden able distance, and the minimum broaden able distance between the sample and all the already existing class centers are calculated, and then the class centers corresponding to the minimum distance are compared whether they are consistent with the given class centers [17]. If not, the class centers and weights are adjusted; then, the weighting coefficients and learning rate are dynamically optimized using the differential evolution algorithm under the constraints of minimum training time and minimum error rate; finally, the above process is repeatedly iterated until the requirements are met. The hidden hazards of production safety accidents are divided into two major categories: basic management and on-site management. Basic management hazards are mainly for production and operation unit qualification and license, production safety management organization and personnel, production safety responsibility system, production safety management system, safety operation procedures, education and training, production safety management files, production safety input, emergency rescue, special equipment basic management, occupational health basic management, related party basic management, other basic management, and other defects. The hidden dangers in the field management category are mainly for the defects in the field management of special equipment, production equipment and facilities, site environment, personnel operation behavior, fire safety, electricity safety, occupational health site safety, limited space site safety, auxiliary power system, related party site management, and other site management.

The sample data are collated to determine the number of feature vectors and the types of recognition. To evaluate the classification performance, the error rate is defined as follows:

(4) $$\begin{array}{c} E_{T}=\frac{N_{m}^{2}}{N_{p}^{2}}, \end{array}$$

where *N*_*m*_ denotes the total number of classification errors, *N*_*p*_ is the total number of classified samples, and *E*_*T*_ is the total error rate. The object element model of the sample data is constructed, and the connection weights and class centers are initialized. The object element model is represented as follows:

(5) $$\begin{array}{c} R_{k}=\left[\begin{array}{ccc} N_{p}^{2} & c_{1} & V_{k1}\\ ... & c_{2} & V_{k2}\\ ... & c_{3} & V_{k3} \end{array}\right]. \end{array}$$

A topological strategy generation system is a computerized strategy implementation system based on polarizability theory and polarizability methods. The system combines the topologic method with the existing artificial intelligence technology, visualization technology, database technology, object-oriented technology, etc. It builds a database based on the normalized description of existing data, finds incompatible problems in the “rule-based,” and generates a “problem base.” Then, based on the topological transformations and inference rules, we use the topological analysis method to build a “topological transformation library,” which is used to generate a strategy library, and finally, use the superiority evaluation method to select the optimal strategy and provide the best solution for decision-makers.

**Figure 2:**
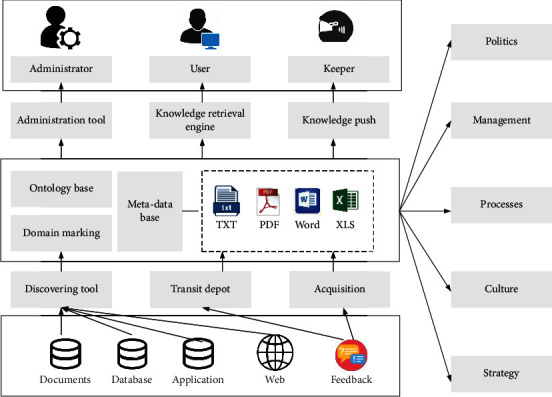
Block diagram of the formal system of topological information-knowledge-strategy.

The topological data mining method is a mining theory and method used to discover the law of data change, to mine the knowledge of change to provide a basis for solving contradictory problems. The research on mining transformations, the role of transformations, and their rules have just started and has a large development space. However, the preliminary research results have shown that combining topology with data mining will greatly expand the existing data mining theories and techniques and provide new ideas for solving the contradictory problems existing in data processing, as shown in Figure 2.

Many problems encountered in daily life are composed of several factors that affect each other. In optimization problems, some of these factors are extracted as optimization objectives, but because their mutual influence is not all positive incentives, often the rest of the objectives will be degraded when a certain objective is improved, which requires us to make trade-offs among multiple objectives or to achieve a compromise result, which is also the important meaning of multiobjective optimization.

In single-objective optimization problems, the value of the final solution is usually used directly to evaluate the goodness of the solution, while multiobjective optimization differs from single-objective optimization in that it does not result in only one solution but a set of noninferior solutions, any one of which cannot be dominated by the rest of the solutions, so the set of solutions given by multiobjective optimization is nondominated. The decision-maker can choose one of these nondominated solutions as the final solution according to the specific preference of his current model. The chemical park should take into account factors such as the prevailing wind direction, the difference in terrain, the interaction between enterprises' installations, product categories, production processes, mutual supply of materials, public facilities. The zone should be reasonably arranged in terms of functional zoning, taking into account factors such as the prevailing wind direction, terrain height, interaction between enterprises, product categories, production processes, mutual supply of materials, public facilities, emergency relief, etc. Labour-intensive nonchemical enterprises shall not be mixed with chemical enterprises in the same chemical park.

There are two types of methods to solve multiobjective optimization problems, one is to use multiobjective algorithms directly to solve the problem, and the other is to transform the multiobjective model into a traditional single-objective model and use some single-objective algorithms to solve this model [18]. The following are several single-objective problem-solving methods.

The linear weighting method is to set the corresponding weight coefficients for each objective according to the importance of each objective, and then multiply each objective value with its corresponding coefficients and finally accumulate them together as the objective of single-objective optimization and turn it into a single objective problem, which is expressed by the following equation:

(6) $$\begin{array}{c} \max f\left(x\right)=\sum_{i=1}^{k}w_{i}f_{i}\left(x^{2}\right). \end{array}$$

This method is simple and easy to understand and is favored by many researchers, but it also has great drawbacks, and the optimization results are too dependent on the setting of the weight coefficients, which is more limited.

**Figure 3:**
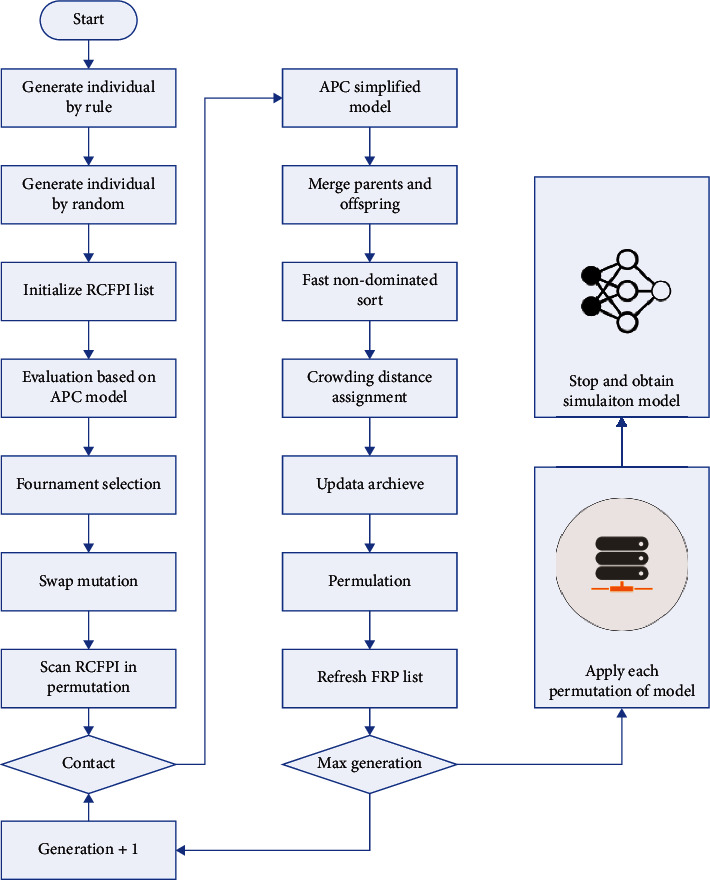
Algorithm flow chart.

The evolutionary algorithm first generates an initialized population, in which the individual individuals in the population consist of codes, each of which represents a solution to the problem. After that, the population is selected, recombined, and mutated according to the evolutionary algorithm, and a brand-new population is generated. The new population and the initial population are combined for fitness value screening, leaving the top *N* optimal individuals, and *N* is the size of the initial population. This is the iterative process of a single loop, which continues until the conditions for the end of the loop are met. The flowchart of the evolutionary algorithm is shown in Figure 3.

After emergency rescue activities begin, emergency supplies will be consumed at a certain rate. To avoid secondary accidents, it is necessary to guarantee an uninterrupted supply of emergency supplies. For example, in the disposal of hazardous chemical warehouse fire and explosion accidents, once there is a shortage of firefighting materials, it is likely to cause the fire to backfire and cause the situation to become more serious. Therefore, to ensure that the current emergency supplies delivered to the accident site can fully support the arrival of the next batch of emergency supplies, there can be no interruption of emergency supplies.

## 4. Construction of Domino Accident Prevention and Control System in Chemical Industry Park

In addition to the three basic components of a general decision support system: user interface, model, and database, the human factor (human interaction, human decision making, etc.) also plays an undeniable role in the decision-making process of specific problems [19]. Decision support systems can be classified into three types from the user use perspective: passive, active, and cooperative. Passive decision support systems facilitate the decision-making process but do not provide recommendations or solutions; active decision support systems provide recommendations or solutions; collaborative decision support systems provide a set of recommendations to be refined or modified by the user later; and from the perspective of how the system works, there are five types: model-driven, data-driven, communication-driven, document-driven, and knowledge-driven. A well-designed and efficient decision support system is generally user-friendly, accessible, widely used, and accurate, and it can help users analyze useful information from the raw data input and make more accurate judgments when they are faced with several choices and decisions that are difficult to specify in advance. It both enhances the efficiency of users' thinking and problem-solving, considering the differences of different solutions, and saves them money and time. Emergency rescue materials and equipment security measures are important material support for emergency rescue and disposal of emergencies in order to further improve the emergency rescue materials and equipment reserves, strengthen the management of emergency materials and equipment, improve the unified deployment of materials and security capacity, for the prevention and disposal of various types of sudden security incidents to provide important protection, according to the “division of labor, unified deployment, preparedness” requirements, especially the development of this measure.

**Figure 4:**
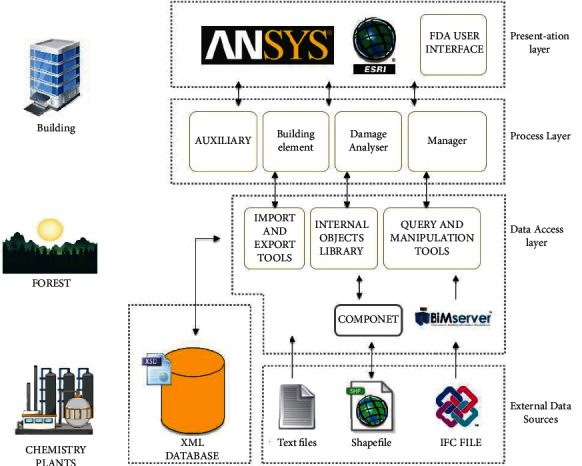
System architecture diagram.

As shown in Figure 4, they all emphasize more the theoretical sense of domino accident risk evaluation and consequence evaluation, ignoring the connection with the actual production situation in the chemical industry. In the actual industrial production process, personnel negligence, improper operation, and other unfavorable factors may also lead to domino accidents. These influencing elements are not included in the simulation calculation of the software, and the implementation of each function only relies on the data of environment, equipment, and materials, so the potential risk of domino is underestimated, and the root cause of accidents is not accurately located.

The evaluation of the consequences of domino accidents in this software only considers the safety level and does not predict the possible impact of the accident on the surrounding environment, which is not conducive to the development of environmental protection. At the same time, the simulation technology of this software is limited and can only simulate secondary domino accident scenarios, lacking the identification and construction of a complete domino accident chain, and there are errors in the calculation of domino accident probability. Enterprises cannot initially grasp the potential domino risks, and enterprises with lower risks do not need to complete the whole process of complex risk evaluation.

The system mainly realizes data exchange between database and application layer module by reading and writing database, etc.; various model algorithms, including the Monte Carlo algorithm, provide support for data result processing in the application layer. The emergency rescue decision support system uses technologies such as natural language processing, text recognition, machine learning, and graph database to fuse data through knowledge modeling, knowledge acquisition, knowledge fusion, and knowledge reasoning links to build an emergency rescue knowledge map, realize functions and services such as knowledge visualization, intelligent search, intelligent question and answer, document management, relationship mining, reasoning management, and map updating, and provide basic support to business systems such as risk identification, plan preparation, emergency drills, and emergency rescue in emergency management. Since the domino effect environmental risk evaluation involves a large amount of data including enterprise information, device information, environmental information, material information, etc., the system mainly adopts sqlsever2012 database to realize continuous storage.

**Figure 5:**
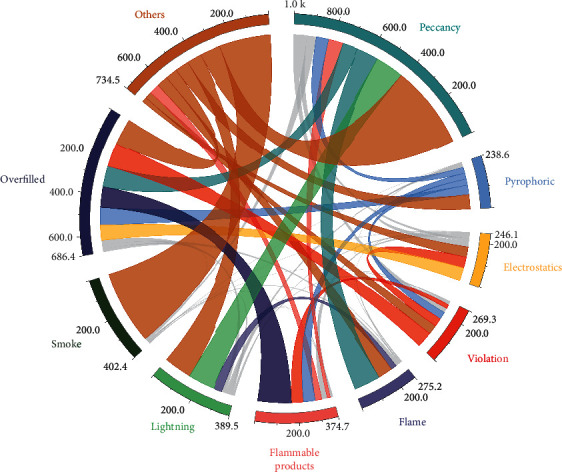
Domino accident cause statistics.

The database of the DOMIRISK system consists of two major components: the basic information database and the case database [20]. Through the summary, we found that the data involved in the domino effect environmental risk evaluation mainly revolves around five aspects: environment, device, material, enterprise, and domino effect risk evaluation results, so the basic information database is designed to contain five subdatabases: material information, device information, environment information, enterprise information, and domino effect risk evaluation results. In addition, due to the lack of resources for summarizing historical domino accidents, a case base for continuously summarizing historical domino accidents is also designed in the DOMIRISK system, as shown in Figure 5.

Direct extension refers to the physical effects generated by the initial accident that directly cause damage to the target equipment, resulting in a domino accident. The chain of accidents containing only the initial accident and the second accident is called “primary” domino accident; if there are three accidents in the chain, it is called a “secondary” domino accident, and the number of levels increases with the growth of the accident chain. Indirect expansion means that the occurrence of the initial accident does not directly cause a domino accident but may be caused by the initial accident scenario that the target unit is out of control, which leads to the occurrence of secondary accidents. In general, the indirect extension of the domino effect is a secondary derivative event triggered by accident, which belongs to the scope of emergency management and is not specifically studied in this paper.

## 5. Results Analysis

### 5.1. Analysis of the Performance Results of the Topological Neural Network Algorithm

**Figure 6:**
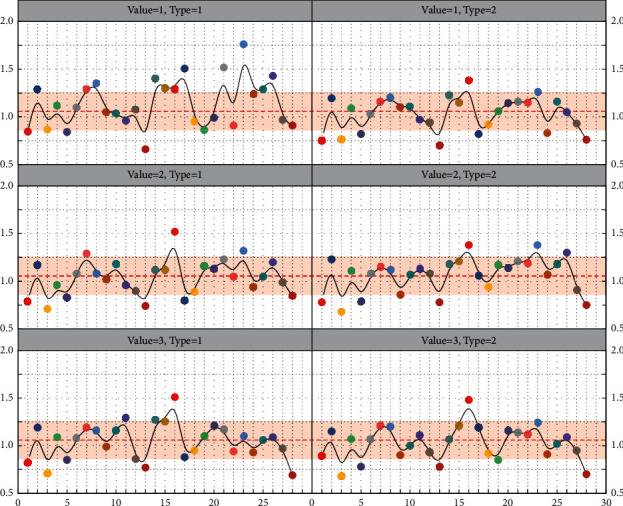
Data test results.

To verify the effectiveness of the DE-ENN algorithm, the Iris dataset and Wine dataset from the UCI database were selected for testing and compared with the BP algorithm, the LVQ algorithm, and the standard ENN algorithm without DE optimization. The training and testing samples required for the simulation tests are generated randomly in proportion. The learning rates of BP, LVQ, and ENN are 0.5, 0.01, and 0.1, respectively, and the simulation results are shown in Figure 6. The number of errors for the BP algorithm is 3 and 4, the number of errors for the LVQ is 2 and 4, the number of errors for the ENN is 1 and 3, and the number of errors for the DE-ENN algorithm is 1 for both the Iris and Wine tests. These results show that DE-ENN significantly outperforms BP and LVQ methods in terms of prediction accuracy and that DE-ENN also has better recognition results than ENN without any optimization for the more complex Wine dataset.

To further verify the prediction performance and generalization ability of the algorithm, this chapter uses a 10-fold cross-validation method to continue the experiment and repeats 10 times to get the average of the results. Compared with BP and LVQ algorithms, the DE-ENN algorithm has a simpler structure, shorter training time, and lower training error rate, and it has a greater advantage in prediction results. In addition, compared with ENN, DE-ENN increases the learning time by 2.8s and 4.4s, respectively, but has a significant improvement in training accuracy and more accurate prediction results, which indicates that the DE-ENN algorithm not only inherits the advantages of simple structure and efficient training of ENN but also has a significant improvement in prediction accuracy. These results show that the algorithm has excellent performance on the one hand and strong generalization ability on the other. Accident handling refers to the operation process of quickly rescuing people, isolating faulty equipment, and adjusting the operation mode in order to quickly resume normal operation in case of an emergency or accident that endangers personal safety or property safety in daily production and life. Such as traffic accident handling, production accident handling, fire accident handling, equipment accident handling, power accident handling, etc.

For data testing, two sets of data from each of the samples corresponding to the risk levels are selected as test samples. Then the method is compared with existing methods, and to ensure effectiveness, each method is tested 10 times using the same training and testing samples, and then averaged. Among them, the feedback gain factor of the Elman network is taken as 0.3, the learning rates of LVQ, BP, and ENN are taken as 0.05, 0.02, and 0.1, respectively, and the crossover factor in the DE-ENN method is taken as CR = 0.3 and the scaling factor *F* = 1.2.

**Figure 7:**
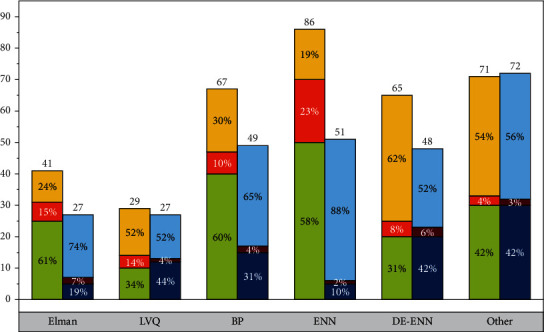
Comparison of the prediction effect of various methods.

From Figure 7, the DE-ENN-based method is the same as other methods such as the Elman method, LVQ method, BP neural network, and ENN method in terms of prediction accuracy, and all of them can have better prediction results. In terms of the number of iterations, the Elman method has the highest number of iterations, which is more than 1000, while the LVQ and BP methods are 28.6 and 243.8, respectively, and the DE-ENN has only 2 iterations, which greatly simplifies the execution process of the algorithm.

In terms of training time, the DE-ENN algorithm takes only 3.8 s to complete, which has a greater advantage compared with Elman, LVQ, and BP; although ENN takes the shortest time among these methods, DE-ENN has a significantly better training error rate and prediction accuracy than ENN with only 2.8 s increase in training time, which indicates that DE-ENN is more efficient than other methods in terms of the training process and prediction results. At the same time, compared with other methods, DE-ENN's eigenvalue classification boundary is clearly defined at the beginning, i.e., more specialized knowledge is obtained before training, which makes it possible to produce more meaningful results at the end of training. On the other hand, the method is highly adaptive to new and important information, easily acquiring the knowledge therein and continuously improving its classification performance.

The parameters of its actual operation are continuously changing and uncertain, in which some noise and uncertainty are inevitably introduced. The generation of errors includes environmental factors, instrument measurement errors, human errors, etc., which can lead to uncertainty and errors in the parameters. Considering the interference of this noisy and uncertain information, a random error of 0%–30% was added to all data to test the fault tolerance of DE-ENN. In this chapter, based on the previous tests, random errors were added to the training samples by error rates of ±0%, ±5%, ±10%, ±15%, ±20%, ±25%, and ±30% in that order, and the different identification methods were tested again, and the results were averaged by testing 10 times.

### 5.2. Analysis of the Results of the Prevention and Control System

In the actual production process, chemical fiber manufacturers will set up a series of safety devices and protective measures to avoid, prevent, and control the occurrence of ignition sources and ignition events, to reduce the probability of ignition after the leakage of combustible materials. These safety devices and protective measures are also called safety barriers, which are classified into process control barriers, ignition source isolation barriers, safety monitoring barriers, fire prevention barriers, and safety management barriers according to the nature of the protective object, the purpose, and conditions of use, and the place of use. Since the above basic ignition probabilities obtained from statistical data or simplified calculation models can not take into account the actual engineering safety barriers' suppression of ignition sources, they can be used to reduce the probability of ignition. Since the base ignition probabilities obtained from the above-mentioned statistical data or simplified calculation models do not consider the suppression effect of safety barriers on ignition sources in actual engineering, which may increase the probability of ignition of combustible materials, a safety barrier compensation factor is proposed in this paper to correct for it.

**Figure 8:**
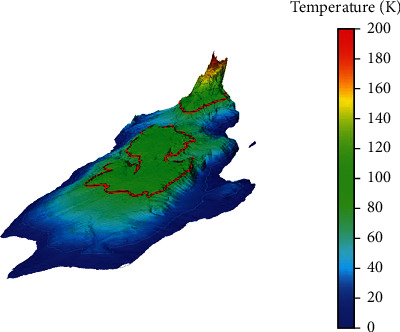
Large storage tank fire gets to control time.

Whether the initial fire incident can extend and lead to a second incident in the target equipment is directly related to the fire control effectiveness of the ignition equipment and the degree of failure of the target equipment. Correct and effective fire emergency measures will affect the damaging effect of fire heat radiation on the target equipment, reduce the intensity of heat radiation received by the target equipment to below the critical threshold, and thus avoid the occurrence of domino accidents. At the early stage of fire accidents, chemical fiber production enterprises mainly used fire detection and alarm system and fixed fire protection system for early warning and rapid response disposal. Whether the two can function effectively in time is the key factor in deciding whether the target equipment occurs secondary story, which can be determined by two key times, the time when the fire of the burning equipment is controlled and the time when the target equipment fails, as shown in Figure 8.

**Figure 9:**
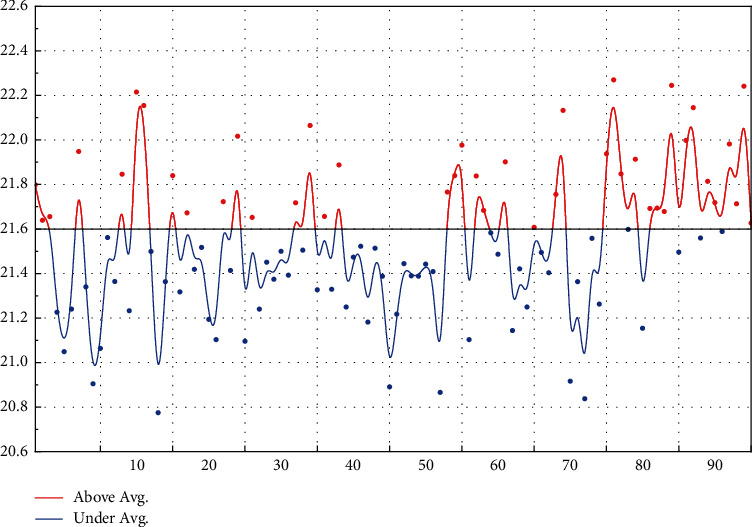
Calculation results.

That is the number of feasible solutions obtained by running each algorithm 30 times on each test instance in four environments, where the better value is bolded. As can be seen from Figure 9, HNSGA-II can explore a significant number of feasible solutions in all four environments; MOEA/D-IEpsilon can only find a small number of feasible solutions; NSGA-II can get a few feasible solutions in environments 1 and 3 but can hardly find feasible solutions in environments 2 and 4; CCMO is difficult to find feasible solutions in all four environments.

Combining the results of the two algorithms, the important nodes of the accident chain network in this chemical park are 3, 8, 22, 25, 26, 35, 36, 37, 57, 60, i.e., the misoperation is likely to cause the malfunction of the production process conditions, as well as other disaster-causing factors leading to reactor failure, pipeline breakage, vessel and tank rupture, etc., which will lead to the occurrence of fire, explosion and leakage accidents, and the propagation and escalation of accidents in the chemical park. Eventually, it will cause serious consequences such as casualties and economic losses. Therefore, in the whole chemical park safety production process, while improving the staff's awareness of prevention and professional skills, it is also necessary to focus on monitoring the production process conditions, production equipment and devices, pipelines, storage tanks, and containers in the park to prevent and reduce the possibility of accidents occurring and spreading in the park. Meanwhile, the results are consistent with the high frequency of accident causes, accident types, and accident consequences in the accident statistics of chemical enterprises, which verifies the accuracy of the node importance evaluation method proposed in this paper.

The experimental results show that with the increasing scale of the problem, HNSGA-II has a much better ability to explore feasible solutions than the other three algorithms and has better stability. This is because MOEA/D-IEpsilon can dynamically adjust the value of epsilon by the proportion of feasible solutions, thus quickly crossing the larger infeasible region and moving toward the feasible domain, but the feasible domain of the multiple-constraint multiobjective optimization model in this paper is quite tiny due to the extremely harsh nature of the continuous consumption constraints, resulting in most of the individuals in the population directly crossing the feasible domain; whereas NSGA-II can only explore feasible solutions through its own constraint violation degree, which has very limited effect on the tiny feasible domain; for CCMO, its auxiliary problem is a type of unconstrained optimization problem, and the large number of infeasible solutions generated can hardly provide useful information for the optimization of the main problem; the heuristic correction strategy embedded in HNSGA-II makes full use of the knowledge of the problem itself, which can make infeasible solutions. The heuristic correction strategy embedded in HNSGA-II makes full use of the knowledge of the problem itself and can allow infeasible solutions to quickly approach the feasible solution region through correction, which greatly improves the ability of the algorithm to mine feasible solutions.

## 6. Conclusion

Based on the standard ENN, an ENN classification algorithm based on boundary discriminant projection and improved SAP clustering is proposed. The experimental results show that the classification algorithm significantly improves the classification ability of ENN for complex sample data while retaining the advantage of ENN for low-dimensional samples. The final practical application also shows that the method has better prediction ability for the melt index (MI) of HDPE, and the MDP-ISAP-ENN has better generalization performance and prediction ability than the standard ENN. After the identification of the potential accident chain, the DOMRISK system can also calculate the overall accident probability of each device through the built-in Monte Carlo simulation tool to integrate different accident scenarios. The ranking of the accident probability of each device can provide a more practical basis for the management of process equipment and help managers to identify the risk vulnerabilities in the devices promptly. When the inherent risk value of the plant is high, the inherent risk can be compensated for by increasing the number of fire medical points in the plant to reduce the fire medical response time, thus achieving the purpose of controlling the domino risk in the plant.

## Data Availability

The data used to support the findings of this study are available from the corresponding author upon request.
